# Predicting Health-Related Quality of Life Outcomes Following Major Scoliosis Surgery in Adolescents: A Latent Class Growth Analysis

**DOI:** 10.1177/21925682221126451

**Published:** 2022-09-20

**Authors:** Jack Kerr, Edward Abraham, Amanda Vandewint, Erin Bigney, Jeffrey Hebert, Eden Richardson, Dana El-Mughayyar, Jill Chorney, Ron El-Hawary, PORSCHE Study Group, Rory McPhee, Neil Manson

**Affiliations:** 1 Faculty of Medicine, 7512 Memorial University of Newfoundland, 300 Prince Philip Drive, St. John’s, NL, A1B 3V6, Canada; 2 Department of Surgery, Dalhousie Medicine New Brunswick, Saint John, NB, Canada; 3 Canada East Spine Centre, Saint John, NB, Canada; 4 Saint John Orthopaedics, Saint John, NB, Canada; 5 10068 Horizon Health Network, Saint John, NB, Canada; 6 Faculty of Kinesiology, 3427 University of New Brunswick, Fredericton, NB, Canada; 7 Canadian Spine Outcomes and Research Network, Markham, ON, Canada; 8 Mental Health and Addictions Program, IWK Health Centre, Halifax, NS, Canada; 9 Department of Psychiatry, Dalhousie University, Halifax, NS, Canada; 10 Department of Surgery, IWK Health Centre, Halifax, NS, Canada; 11 McGill University, Montreal, QC, Canada; 12 Stollery Children’s Hospital, Edmonton, AB, Canada; 13 University of Alberta, Edmonton, AB, Canada; 14 McMaster University, Hamilton, ON, Canada; 15 Alberta Children’s Hospital, Calgary, AB, Canada; 16 Children’s Hospital of Eastern Ontario, Ottawa, ON, Canada; 17 Ste. Justine Hospital, Montreal, QC, Canada; 18 3427 University of New Brunswick, Saint John, NB, Canada

**Keywords:** adolescent, scoliosis, trajectory analysis, health-related quality of life

## Abstract

**Study Design:**

Prospective cohort study.

**Objectives:**

To identify patient trajectories of recovery defined by change in health-related quality of life (HRQOL) following surgery for adolescent idiopathic scoliosis (AIS). To explore possible predictors of trajectory membership.

**Methods:**

Adolescent patients scheduled to undergo spinal fusion for AIS were enrolled in the *Post-Operative Recovery following Spinal Correction: Home Experience (PORSCHE)* study. Responses to the Pediatric Quality of Life Inventory–version 4 (PedsQL–4.0) were collected prior to surgery and 4 to 6 weeks, 3, 6, and 12 months post-operatively. Latent class growth analyses identified patient subgroups based on their unique trajectories of physical health (PH) and psychosocial health (PSH) outcomes using the PedsQL–4.0 subscale scores. Predictors included demographic, clinical, and psychosocial factors.

**Results:**

Data from up to 190 patients were included (87.4% female; mean±SD age = 14.6 ± 1.9 years). Three trajectory subgroups were identified for PH and 4 trajectories were found for PSH, with a majority of patients scoring within the established range of healthy adolescents 12 months post-surgery. Increased child and parent pain catastrophizing, child trait anxiety and previous hospitalizations were associated with poorer PH outcomes, whereas increased child and parent pain catastrophizing, child state and trait anxiety, and parent state and trait anxiety were associated with poorer PSH trajectories.

**Conclusions:**

The PH and PSH trajectories identified in this study and the factors associated with their membership may inform surgical decision-making for AIS while facilitating patient and family counselling regarding peri-operative recovery and expectations.

## Introduction

Scoliosis represents a group of medical conditions in which the affected individual has abnormal spinal curvature. Adolescent Idiopathic Scoliosis (AIS) is the most common form of disease presentation, which affects 2% to 4% of adolescents^1‐3^. Previous studies have shown that patients with AIS have restrictions with physical function, which worsen as the spine angle increases^4,5^. Surgery is recommended when progressive AIS exceeds 45 degrees in patients with an immature skeleton or when progression or pain occurs after skeletal maturity^6^. Children who undergo surgery often receive a form of spinal fusion which aims to correct the curvature of their spine and prevent further progression of the condition. In addition to changing the shape of the spine, patients and clinicians often anticipate improved health-related quality of life (HRQOL) after surgery^7,8^. Surgically treated patients are reported to have comparable or slightly lower HRQOL than healthy individuals, and spinal fusion has been found to positively improve HRQOL across all domains in patients with AIS^9‐11^.

There has been extensive research on postoperative pain in patients with AIS, and recent studies have focused on measuring pain at various time points and using trajectory modeling to describe the changes in pain^12‐16^. This is due to the growing recognition that measuring pain at a single time point, such as immediately postoperatively, or using averages of pain scores over time, limits the understanding of the natural course of an individual’s postsurgical pain. Many patients may not fit an “average” recovery trajectory, and this is likely explained by the large variability of clinical outcomes.

While the association between pain and HRQOL has been thoroughly examined, no pediatric study has explored HRQOL trajectory subgroups outside the context of pain, a differentiation which could assist clinical decision-making^15,16^. Furthermore, although previous studies have reported that sex, preoperative pain levels, anxiety sensitivity in children, and parental pain catastrophizing are factors that play a role in shaping pain responses^12–17^, the suitability of these factors as predictors of short- and long-term HRQOL outcomes has yet to be assessed. As such, this study evaluates the various demographic, psychosocial and perioperative factors and their association with postoperative function. The aims of this study were to (1) identify patient cohorts defined by change in HRQOL for both physical and psychosocial health recovery following surgery for AIS, and (2) explore various potential predictors of subgroup membership.

## Methods

### Study Design and Participants

The *Post-Operative Recovery following Spinal Correction: Home Experience (PORSCHE)* study is a multicenter initiative examining prevalence, predictors, and consequences of children’s pain following scoliosis surgery. The PORSCHE study methodology has previously been described in the literature^16,18^. Participants were recruited at 8 hospitals across Canada, including the Izaak Walter Killam Health Centre (Halifax, Nova Scotia), Saint John Regional Hospital (Saint John, New Brunswick), Montreal Children’s Hospital (Montreal, Quebec), Centre Hospitalier Universitaire Sainte-Justine (Montreal, Quebec), Children’s Hospital of Eastern Ontario (Ottawa, Ontario), McMaster Children’s Hospital (Hamilton, Ontario), Stollery Children’s Hospital (Edmonton, Alberta), and Alberta Children’s Hospital (Calgary, Alberta). Questionnaires were completed preoperatively and then at 4 to 6 weeks, 3, 6 and 12 months after surgery.

Eligible participants included children and adolescents aged 10 to 18 years old who were scheduled to undergo spinal fusion and instrumentation for AIS. The indication for surgery was a progressive scoliosis greater than 40 to 45 degrees in skeletally immature patients, and greater than 50 to 55 degrees in skeletally mature patients. Exclusion criteria were: inability to read or speak English, developmental delay that would interfere with completing study measures, or major chronic medical conditions (ASA status III or higher). The PORSCHE study was approved by the respective Research Ethics Boards at all study sites and all initial patient data was prospectively collected with informed consent. The present investigation which uses data from the larger PORSCHE study obtained Horizon Health Network Research Ethics Board approval and no informed consent was required (File #: 2020-2861).

### Measures

#### HRQOL outcomes

##### Pediatric quality of life inventory – version 4 (PedsQL-4.0)

This questionnaire was administered at baseline, and at 4 to 6 weeks, 3, 6 and 12 months postoperatively. This questionnaire measures the child’s general quality of life in 4 categories: physical, emotional, social and school ^19^. Children indicate on a scale of 0 to 4, the degree of problems they encounter with each item. Items are then reverse scored and linearly transformed on a 0 to 100 scale such that higher scores indicate greater quality of life. This scale offers a short administration time to provide an effective measurement of HRQOL for pediatric orthopaedic groups, including patients with scoliosis^19,20^. Summary scores and total scores generated from the PedsQL-4.0 in adolescent patients with idiopathic scoliosis have demonstrated a low-moderate strength correlation with total scores from the Scoliosis Research Society-22 revised questionnaire which is a disease-specific tool (Spearman correlation coefficients between .26-.37)^20^. PedsQL-4.0 was selected as the HRQOL measure of choice for this study to allow for physical and psychosocial outcome categorization using standardized summary scores. Additionally, use of a generic HRQOL instrument allows for outcome interpretation and comparison within a broader context of pediatric populations with various orthopaedic and non-orthopaedic pathologies^19,20^. Age-appropriate versions were used (validated versions are for ages 8 to 12 and 13 to 18 years).

#### Baseline predictors

##### Demographic and clinical information

Collected demographic variables used as predictors included sex, age, length of hospital stay, highest magnitude of spinal curvature and history of previous surgeries, hospitalizations, and prematurity at birth.

##### State-trait anxiety inventory

This questionnaire evaluates both the state and trait level of anxiety of the child using the *State-Trait Anxiety Inventory – Child Form (STAIC)* or the parent using the *State-Trait Anxiety Inventory – Parent Form (STAIP)*^21,22^*.* The child or parent is presented with statements of how people describe themselves and are asked to evaluate on a scale of 1 to 3 for the STAIC or a scale of 1 to 4 for the STAIP, whether or not they agree with the statement at the current moment, as well as in general. Some items are reverse scored so that a greater score indicates greater anxiety with scores ranging from 20 to 60 using the STAIC for both state and trait anxiety or 20 to 80 using the STAIP.

##### Pain catastrophizing scale

This questionnaire measures children’s thoughts and feelings in response to pain using the *Pain Catastrophizing Scale – Child Form (PCS-C)* or parent’s thoughts and feelings in response to their child’s pain using the *Pain Catastrophizing Scale – Parent Form (PCS-P)* ^23,24^. Children rate their anxiety responses to pain while parent’s rate their responses to their child’s pain on a 5-point, 13 item, Likert-type scale ranging from, “Not at all” to “Extremely”. Items are summed to yield total scores ranging from 0 to 52 with higher scores indicating a greater amount of catastrophizing in response to pain.

### Data Analysis

#### Identification of trajectories

All analyses were conducted with Stata 16.1 software (StataCorp, College Station, TX, USA). HRQOL outcomes measured at preoperative baseline and 4 to 6 weeks, 3 months, 6 months, and 12 months after surgery were used to assign each patient to a trajectory subgroup with latent class growth analysis. This is a specialized application of finite mixture modeling that provides an empirical method of identifying meaningful subgroups of patients, based on their patterns of change (ie, trajectories) over time^25,26^.

Patients with missing outcome scores at baseline and those without a minimum of 1 follow-up outcome measure were excluded. Latent class growth models handle missing data with maximum likelihood estimation, resulting in asymptotically unbiased parameter estimates when data are missing at random^25^.

Single class models were first generated with number of classes and the complexity of polynomial distributions (eg, linear, quadratic, cubic) increased until optimal models were identified. Initial model specification was based on the Bayesian information criterion and clinical judgment. Models were subsequently evaluated with 4 diagnostic criteria^25,26^: (1) an average posterior probability of individual group membership of .7 or greater for each group; (2) close correspondence between the estimated probability of group membership and the proportion of participants assigned to each subgroup; (3) reasonably tight confidence intervals around estimated group membership probabilities and (4) minimum odds of correct classification >5 for each subgroup^27^.

#### Identification of outcome predictors

Predictors of trajectory subgroup membership were investigated using multinomial regression models with robust standard errors. Model results were reported with relative risk ratios (RRR) and 95% confidence intervals (95% CI). For the *State-Trait Anxiety Inventory* and *Pain Catastrophizing Scale* scores, the RRR was reported per 1 standard deviation unit of change. A consistent reference subgroup was selected for comparisons between the different physical health trajectories identified as well as the psychosocial health trajectories. Each latent class growth model yielded 1 subgroup with large changes in physical or psychosocial health. Consequently, we undertook a post hoc analysis to explore the marginal predicted probabilities of membership in these subgroups for each significant predictor.

## Results

Data from 207 patients were assessed for eligibility. Data from 19 participants for the physical health analysis and 17 participants for the psychosocial health analysis were excluded for not having completed a minimum of 1 follow-up outcome measure. In total, data from up to 190 patients were included in 1 or more analysis. Preoperative demographic, psychosocial, and surgical information is presented in Table 1 and Table 2 for the physical health trajectory groups and psychosocial health trajectory groups, respectively.

**Table 1.** Baseline Demographics, Questionnaire Scores and Surgical Details by Physical Health Trajectory Groups.

Variable	‘Moderate Start, Minor Decline, High Finish’	‘High Start, Major Decline, High Finish’	‘High Start, Moderate Decline, High Finish’	Overall
Child age in years at baseline [mean ± SD]	14.9 ± 1.9, N = 35	14.5 ± 1.7, N = 101	14.8 ± 2.1, N = 41	**14.6 ± 1.8, N = 177**
Female sex (percent of total)	33 (91.7%), N = 36	93 (86.9%), N = 107	37 (82.2%), N = 45	**163 (86.7%), N = 188**
Has had previous surgery (percent of total)	10 (29.4%), N = 34	38 (36.2%), N = 105	12 (26.7%), N = 45	**60 (32.6%)**, **N = 184**
Has been previously hospitalized (percent of total)	21 (58.3%), N = 33	42 (40.0%), N = 105	13 (29.5%), N = 44	**76 (41.8%), N = 182**
Has a history of prematurity (born < 36 weeks) (percent of total)	3 (9.1%), N = 33	10 (9.7%), N = 103	3 (6.8%), N = 44	**16 (8.9%), N = 180**
Number of spine levels [mean ± SD]	10.3 ± 2.8, N = 30	10.8 ± 2.1, N = 84	11.0 ± 2.2, N = 36	**10.7 ± 2.3, N = 150**
Previously used a brace (percent of total)	10 (43.5%), N = 23	27 (33.3%), N = 81	10 (35.7%), N = 28	**47 (35.6%), N = 132**
Highest curve magnitude in degrees [mean ± SD]	64.8 ± 14.8, N = 27	65.6 ± 12.9, N = 98	65.3 ± 14.9, N = 38	**65.3 ± 13.6, N = 163**
Had surgery with a posterior approach (percent of total)	32 (100%), N = 32	93 (96.9%), N = 96	38 (92.7%), N = 41	**163 (96.4%), N = 169**
Upper instrumented vertebra (UIV; percent of total)	T1-T5: 25 (83.3%)	T1-T5: 80 (94.1%)	T1-T5: 34 (89.5%)	**N = 153**
	T6-T8: 1 (3.3%)	T6-T8: 2 (2.4%)	T6-T8: 4 (10.5%)	
	T9-T12: 4 (13.3%)	T9-T12: 3 (3.5%)	T9-T12: 0 (.0%)	
Lower instrumented vertebra (LIV; percent of total)	T10-T12: 2 (6.7%)	T10-T12: 9 (10.5%)	T10-T12: 6 (16.7%)	**N = 152**
	L1-L3: 17 (56.7%)	L1-L3: 63 (73.3%)	L1-L3: 20 (55.6%)	
	L4-L5: 11 (36.7%)	L4-L5: 14 (16.3%)	L4-L5: 10 (27.8%)	
Apex of instrumented curve (percent of total)	T5-T6: 0 (.0%)	T5-T6: 3 (3.5%)	T5-T6: 2 (5.4%)	**N = 152**
	T7-T10: 25 (83.3%)	T7-T10: 79 (92.9%)	T7-T10: 33 (89.2%)	
	T11-L1: 5 (16.7%)	T11-L1: 3 (3.5%)	T11-L1: 2 (5.4%)	
Lenke type (percent of total)	1: 9 (42.9%)	1: 37 (41.1%)	1: 17 (47.2%)	**N = 147**
	2: 2 (9.5%)	2: 21 (23.3%)	2: 6 (16.7%)	
	3: 5 (23.8%)	3: 14 (15.6%)	3: 7 (19.4%)	
	4: 2 (9.5%)	4: 10 (11.1%)	4: 2 (5.6%)	
	5: 2 (9.5%)	5: 3 (3.3%)	5: 2 (5.6%)	
	6: 1 (4.8%)	6: 5 (5.6%)	6: 2 (5.6%)	
UIV Anchor Type (percent of total)	Hook: 0 (.0%)	Hook: 0 (.0%)	Hook: 1 (2.7%)	**N = 154**
	Screw: 25 (96.2%)	Screw: 80 (87.9%)	Screw: 35 (94.6%)	
	Hybrid: 1 (3.8%)	Hybrid: 11 (12.1%)	Hybrid: 1 (2.7%)	
Length of Hospital Stay in Days [mean ± SD]	6.3 ± 2.1, N = 36	5.6 ± 1.2, N = 107	7.3 ± 11.9, N = 45	**6.1 ± 6.0, N = 188**
Duration of Surgery in Minutes [mean ± SD]	342.8 ± 148.2, N = 30	333.9 ± 110.4, N = 72	308.5 ± 111.2, N = 37	**329.1 ± 119.5, N = 139**
Parent State Anxiety Score (STAIP) [mean ± SD]	50.4 ± 12.8, N = 30	46.0 ± 10.8, N = 93	44.8 ± 12.4, N = 42	**46.5 ± 11.7, N = 165**
Parent Trait Anxiety Score (STAIP) [mean ± SD]	39.1 ± 11.0, N = 31	37.6 ± 8.6, N = 91	38.0 ± 10.1, N = 41	**38.0 ± 9.4, N = 163**
Child State Anxiety Score (STAIC) [mean ± SD]	38.2 ± 6.5, N = 31	34.6 ± 6.6, N = 93	34.6 ± 7.9, N = 39	**35.3 ± 7.0, N = 163**
Child Trait Anxiety Score (STAIC) [mean ± SD]	40.3 ± 6.8, N = 33	34.4 ± 7.0, N = 96	34.6 ± 6.3, N = 41	**35.6 ± 7.1, N = 170**
Parent Pain Catastrophizing Score (PCS-P) [mean ± SD]	26.9 ± 10.8, N = 30	22.6 ± 8.4, N = 81	21.4 ± 8.7, N = 35	**23.2 ± 9.2, N = 146**
Child Pain Catastrophizing Score (PCS-C) [mean ± SD]	26.0 ± 8.9, N = 34	18.1 ± 9.5, N = 102	17.9 ± 8.1, N = 40	**19.6 ± 9.6, N = 176**

Values are reported as counts (percentage) unless otherwise indicated.

**Table 2.** Baseline Demographics, Questionnaire Scores and Surgical Details by Psychosocial Health Trajectory Groups.

Variable	‘High Start, High Finish’	‘Moderate Start, High Finish’	‘High Moderate Start, High Finish’	‘Moderate Start, Moderate Finish’	Overall
Child Age in Years at Baseline [mean ± SD]	14.6 ± 1.7, N = 75	14.4 ± 1.9, N = 35	14.9 ± 1.8, N = 44	14.5 ± 2.2, N = 25	**14.6 ± 1.9, N = 179**
Female Sex (percent of total)	70 (85.4%), N = 82	31 (88.6%), N = 35	41 (85.4%), N = 48	24 (96.0%), N = 25	**166 (87.4%), N = 190**
Has Had Previous Surgery (percent of total)	29 (36.3%), N = 80	13 (37.1%), N = 35	13 (27.1%), N = 48	5 (21.7%), N = 23	**60 (32.3%)**, **N = 186**
Has Been Previously Hospitalized (percent of total)	30 (38.0%), N = 79	16 (45.7%), N = 35	19 (39.6%), N = 48	11 (50.0%), N = 22	**76 (41.3%), N = 184**
Has a History of Prematurity (born < 36 weeks) (percent of total)	8 (9.8%), N = 82	3 (9.4%), N = 32	3 (6.5%), N = 46	2(8.7%), N = 23	**16 (8.7%), N = 183**
Number of Spine Levels [mean ± SD]	10.6 ± 2.2, N = 51	10.4 ± 2.8, N = 34	11.3 ± 2.2, N = 45	10.4 ± 1.9, N = 19	**10.7 ± 2.3, N = 149**
Previously Used a Brace (percent of total)	18 (41.9%), N = 43	11 (34.4%), N = 32	13 (29.5%), N = 44	7 (58.3%), N = 12	**49 (37.4%), N = 131**
Highest Curve Magnitude in Degrees [mean ± SD]	63.6 ± 12.3, N = 74	72.9 ± 14.2 N = 33	64.1 ± 13.4, N = 47	59.5 ± 12.4, N = 10	**65.4 ± 13.5, N = 164**
Had Surgery with a Posterior Approach (percent of total)	67 (98.5%), N = 68	30 (93.8%), N = 32	45 (95.7%), N = 47	22 (95.7%), N = 23	**164 (96.5%), N = 170**
Upper Instrumented Vertebra (UIV; percent of total)	T1-T5: 49 (90.7%)	T1-T5: 29 (87.9%)	T1-T5: 42 (91.3%)	T1-T5: 19 (100%)	**N = 152**
	T6-T8: 1 (1.9%)	T6-T8: 3 (9.1%)	T6-T8: 2 (4.3%)	T6-T8: 0 (.0%)	
	T9-T12: 4 (7.4%)	T9-T12: 1 (3.0%)	T9-T12: 2 (4.3%)	T9-T12: 0 (.0%)	
Lower Instrumented Vertebra (LIV; percent of total)	T10-T12: 10 (18.5%)	T10-T12: 5 (14.7%)	T10-T12: 0 (.0%)	T10-T12: 2 (10.5%)	**N = 153**
	L1-L3: 31 (57.4%)	L1-L3: 26 (76.5%)	L1-L3: 30 (65.2%)	L1-L3: 12 (63.2%)	
	L4-L5: 13 (24.1%)	L4-L5: 3 (8.8%)	L4-L5: 16 (34.8%)	L4-L5: 5 (26.3%)	
Apex of Instrumented Curve (percent of total)	T5-T6: 5 (9.4%)	T5-T6: 0 (.0%)	T5-T6: 0 (.0%)	T5-T6: 0 (.0%)	**N = 152**
	T7-T10: 43 (81.1%)	T7-T10: 33 (97.1%)	T7-T10: 43 (93.5%)	T7-T10: 19 (100%)	
	T11-L1: 5 (9.4%)	T11-L1: 1 (2.9%)	T11-L1: 3 (6.5%)	T11-L1: 0 (.0%)	
Lenke Type (percent of total)	1: 30 (41.7%)	1: 15 (48.4%)	1: 17 (36.2%)	1: 0 (.0%)	**N = 151**
	2: 11 (15.3%)	2: 5 (16.1%)	2: 13 (27.7%)	2: 1 (100%)	
	3: 16 (22.2%)	3: 3 (9.7%)	3: 8 (17.0%)	3: 0 (.0%)	
	4: 3 (4.2%)	4: 4 (12.9%)	4: 6 (12.8%)	4: 0 (.0%)	
	5: 6 (8.3%)	5: 1 (3.2%)	5: 3 (6.4%)	5: 0 (.0%)	
	6: 6 (8.3%)	6: 3 (9.7%)	6: 0 (.0%)	6: 0 (.0%)	
UIV Anchor Type (percent of total)	Hook: 0 (.0%)	Hook: 0 (.0%)	Hook: 1 (2.1%)	Hook: 0 (.0%)	**N = 154**
	Screw: 61 (89.7%)	Screw: 27 (90.0%)	Screw: 45 (95.7%)	Screw: 8 (88.9%) Hybrid: 1	
	Hybrid: 7 (10.3%)	Hybrid: 3 (10.0%)	Hybrid: 1 (2.1%)	(11.1%)	
Length of Hospital Stay in Days [mean ± SD]	6.6 ± 8.9, N = 82	5.7 ± 1.3, N = 35	5.5 ± 1.3, N = 48	6.1 ± 2.2, N = 25	**6.2 ± 6.0, N = 188**
Duration of Surgery in Minutes [mean ± SD]	272.8 ± 96.2, N = 42	412.7 ± 107.4, N = 31	372.2 ± 107.9, N = 46	258.2 ± 85.3, N = 18	**335.9 ± 118.0, N = 137**
Parent State Anxiety Score (STAIP) [mean ± SD]	42.7 ± 11.8, N = 71	49.6 ± 11.6, N = 30	49.0 ± 11.8, N = 42	48.5 ± 10.0, N = 22	**46.3 ± 11.8, N = 165**
Parent Trait Anxiety Score (STAIP) [mean ± SD]	36.3 ± 9.1, N = 70	38.2 ± 10.3, N = 32	38.8 ± 8.9, N = 41	41.9 ± 9.4, N = 22	**38.0 ± 9.5, N = 164**
Child State Anxiety Score (STAIC) [mean ± SD]	32.7.2 ± 6.0, N = 66	36.7 ± 7.2, N = 30	35.5 ± 6.6, N = 46	39.9 ± 7.5, N = 22	**35.2 ± 7.0, N = 164**
Child Trait Anxiety Score (STAIC) [mean ± SD]	30.5 ± 5.7, N = 70	37.7 ± 6.0, N = 31	38.0 ± 6.1, N = 47	42.0 ± 5.4, N = 23	**35.4 ± 7.2, N = 171**
Parent Pain Catastrophizing Score (PCS-P) [mean ± SD]	21.3 ± 9.0, N = 63	23.9 ± 7.8, N = 29	25.3 ± 10.6, N = 36	24.7 ± 8.1, N = 19	**23.2 ± 9.2, N = 147**
Child Pain Catastrophizing Score (PCS-C) [mean ± SD]	15.6 ± 8.7, N = 76	22.7 ± 9.4, N = 34	21.1 ± 9.2, N = 45	24.0 ± 8.4, N = 23	**19.4 ± 9.5 N = 178**

Values are reported as counts (percentage) unless otherwise indicated.

### HRQOL Outcome Trajectories

All latent class growth models achieved adequate performance according to our predefined diagnostic criteria (Table 3). Average HRQOL outcomes, stratified by trajectory group, are presented in Supplementary Table 1.

**Table 3.** Latent Class Growth Model Diagnostics for HRQOL Physical Functioning and Psychosocial Functioning Models.

	Average posterior probability^1^	Estimated membership % (95% CI)	Assigned membership %	Odds of correct classification^2^
Physical functioning trajectory groups (N = 188)				
‘Moderate Start, Minor Decline, High Finish’	.86	19.3 (15.2, 23.4)	19.1	26.5
‘High Start, Major Decline, High Finish’	.86	54.5 (47.8, 61.2)	56.9	5.0
‘High Start, Moderate Decline, High Finish’	.84	26.1 (20.1, 32.1)	23.9	15.3
Psychosocial functioning trajectory groups (N = 190)				
‘High Start, High Finish’	.91	42.9 (37.5, 48.3)	43.2	13.4
‘Moderate Start, High Finish’	.73	17.5 (11.8, 23.2)	18.4	13.0
‘High Moderate Start, High Finish’	.86	26.1 (21.6, 20.6)	25.3	17.0
‘Moderate Start, Moderate Finish’	.93	13.4 (10.4, 16.4)	13.2	79.6

1: minimum threshold = .70; 2: minimum threshold = 5.0.

#### HRQOL – physical health

**Figure 1.**
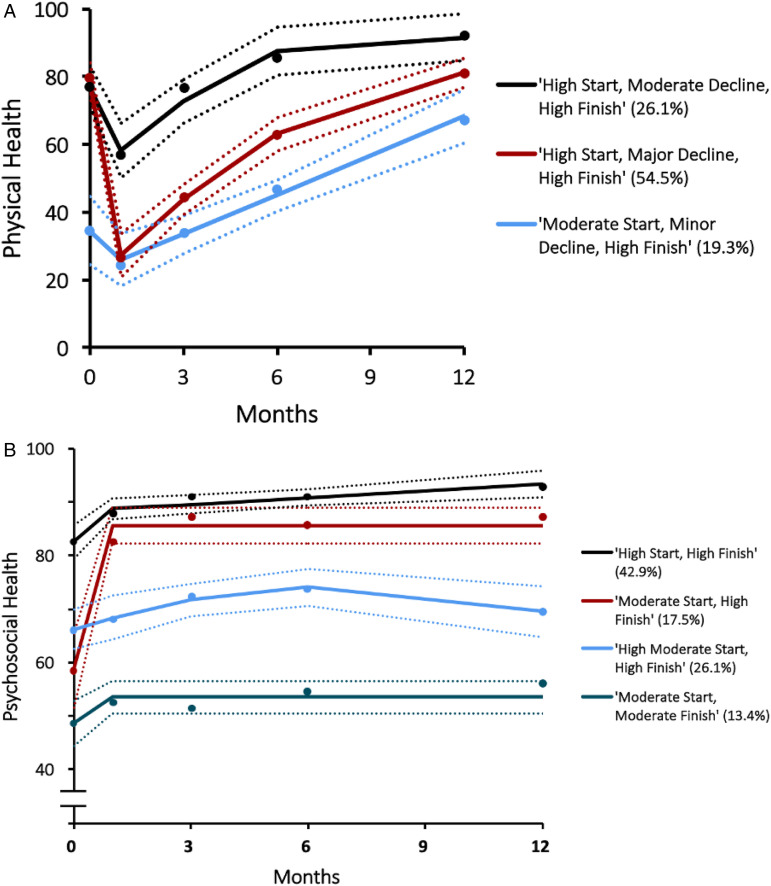
HRQOL – Trajectory groups with prevalence estimates. (A) Physical functioning trajectory groups (N = 188). Point estimates are average HRQOL - physical functioning scores; dotted lines represent 95% confidence intervals. (B) Psychosocial functioning trajectory groups (N = 190). Point estimates are average HRQOL – psychosocial functioning scores; dotted lines represent 95% confidence intervals.

The HRQOL – physical health trajectory model identified 3 trajectory subgroups with quadratic or linear polynomial distributions (Figure 1A). The ‘moderate start, minor decline, high finish’ group (linear distribution) comprised 19.3% of patients who experienced large improvements in HRQOL, with an upward trajectory resulting in nearly double the HRQOL by 12 months. Patients in the ‘high start, major decline, high finish’ group (quadratic distribution, 54.5% of population) experienced drastic short-term decreases in HRQOL, but were able to return to baseline at 12 months. Patients in the ‘high start, moderate decline, high finish’ group (quadratic distribution, 26.1% of population) experienced gradual improvements in HRQOL that remained fairly stable over the course of follow-up. All 3 trajectories at 12 months scored within the established range of healthy adolescents (mean score ± SD = 84.41 ± 17.26), suggesting that the majority of patients achieved this benchmark 1 year following surgery^27^.

#### HRQOL – psychosocial health

The HRQOL – psychosocial health trajectory model identified 4 trajectory groups with linear or quadratic polynomial distributions (Figure 1B). The ‘high start, high finish’ group (linear distribution) included 42.9% of patients who started and maintained scores within a healthy baseline range. Patients in the ‘moderate start, high finish’ group (quadratic distribution, 17.5% of population) experienced rapid, excellent and sustained improvements in psychosocial functioning. The ‘high moderate start, high finish’ group (linear distribution) was comprised of 26.1% of patients who started just below the healthy baseline range, and slightly improved over the course of 12 months in order to finish inside the healthy baseline range. Patients in the ‘moderate start, moderate finish’ group (linear distribution, 13.4% of population) exhibited a maintained moderate psychosocial HRQOL score throughout the follow-up period. At 12 months, 3 out of 4 trajectories scored within the established range of healthy adolescents (mean score ± SD = 82.38 ± 15.51), suggesting that the majority of patients fell into this range 1 year following surgery ^27^.

### Baseline Predictors Associated With Trajectory Membership

Table 4 and Table 5 show the various predictors associated with trajectory membership for physical health and psychosocial health, respectively. The subgroups with the highest physical and psychosocial health scores across time were selected as the reference groups in the case of each trajectory model.

**Table 4.** Predictors Associated With Trajectory Membership for Physical Health Trajectory Groups.

	‘Moderate Start, Minor Decline, High Finish’	‘High Start, Major Decline, High Finish’	‘High Start, Moderate Decline, High Finish’
Child sex	2.38 (.58, 9.75)	1.44 (.55, 3.72)	Ref
Age	1.02 (.78, 1.32)	.90 (.73, 1.12)	Ref
Length of stay	.98 (.95, 1.02)	.87 (.72, 1.06)	Ref
Previous surgery	1.14 (.42, 3.09)	1.56 (.72, 3.38)	Ref
Previous hospitalization	**4.17 (1.59, 10.93)**	1.59 (.74, 3.39)	Ref
Prematurity	1.37 (.26, 7.28)	1.47 (.38, 5.64)	Ref
Highest curve magnitude	1.00 (.96, 1.04)	1.00 (.97, 1.03)	Ref
Parent pain catastrophizing*	**1.83 (1.10, 3.05)**	1.15 (.76, 1.73)	Ref
Child pain catastrophizing*	**2.43 (1.54, 3.84)**	1.02 (.70, 1.48)	Ref
Parent state anxiety*	1.66 (.96, 2.87)	1.11 (.76, 1.62)	Ref
Parent trait anxiety*	1.13 (.68, 1.87)	.96 (.66, 1.40)	Ref
Child state anxiety*	1.64 (.98, 2.75)	1.00 (.64, 1.55)	Ref
Child trait anxiety*	**2.39 (1.47, 3.89)**	.97 (.67, 1.41)	Ref

All values are relative risk ratios with 95% CIs to the reference group for 1 change in unit unless otherwise signified. Bolded values represent significance (*P* < .05).

*Signifies the relative risk ratio for 1 change in standard deviation.

**Table 5.** Predictors Associated With Trajectory Membership for Psychosocial Health Trajectory Groups.

	‘Moderate Start, Moderate Finish’	‘Moderate Start, High Finish’	‘High Moderate Start, High Finish’	‘High Start, High Finish’
Child sex	4.11 (.51, 33.52)	1.33 (.40, 4.46)	1.00 (.37, 2.76)	Ref	
Age	.97 (.73, 1.28)	.94 (.75, 1.17)	1.08 (.89, 1.32)	Ref	
Length of stay	.99 (.95, 1.03)	.96 (.86, 1.07)	.88 (.70, 1.12)	Ref	
Previous surgery	.49 (.16, 1.46)	1.04 (.46, 2.37)	.65 (.30, 1.43)	Ref	
Previous hospitalization	1.63 (.63, 4.24)	1.38 (.61, 3.08)	1.07 (.51, 2.24)	Ref	
Prematurity	.88 (.17, 4.49)	.96 (.24, 3.87)	.65 (.16, 2.57)	Ref	
Highest hurve magnitude	.97 (.93, 1.02)	**1.06 (1.02, 1.09)**	1.00 (.97, 1.03)	Ref	
Parent pain catastrophizing*	1.48 (.92, 2.39)	1.37 (.91, 2.05)	**1.58 (1.02, 2.46)**	Ref	
Child pain catastrophizing*	**2.77 (1.69, 4.55)**	**2.41 (1.52, 3.83)**	**2.01 (1.30, 3.12)**	Ref	
Parent state anxiety*	**1.68 (1.07, 2.63)**	**1.87 (1.16, 3.02)**	**1.77 (1.16, 2.71)**	Ref	
Parent trait anxiety*	**1.82 (1.13, 2.93)**	1.24 (.78, 1.98)	1.33 (.90, 1.97)	Ref	
Child state anxiety*	**3.05 (1.76, 5.28)**	**1.97 (1.23, 3.16)**	**1.65 (1.08, 2.51)**	Ref	
Child trait anxiety*	**11.19 (5.12, 24.45)**	**4.78 (2.60, 8.79)**	**5.12 (2.77, 9.44)**	Ref	

All values are relative risk ratios with 95% CIs to the reference group for 1 change in unit unless otherwise signified. Bolded values represent significance (*P* < .05).

*Signifies the relative risk ratio for 1 change in standard deviation.

#### HRQOL – physical health

Of note, children with previous hospitalizations were 4 times more likely (RRR[95% CI] = 4.17 [1.59-10.93]) to belong to the ‘moderate start, minor decline, high finish’ trajectory group as compared to the ‘high start, moderate decline, high finish’ trajectory group. Higher scores for child (RRR[95% CI] = 2.43 [1.54-3.84]) and parent (RRR[95% CI] = 1.83 [1.10-3.05]) pain catastrophizing as well as child trait anxiety (RRR[95% CI] = 2.39 [1.47-3.89]) were also more likely to result in membership within the ‘moderate start, minor decline, high finish’ trajectory group as compared to the ‘high start, moderate decline, high finish’ trajectory group. There was no association between remaining predictor variables, in regard to membership in the ‘moderate start, minor decline, high finish’ trajectory group as compared to the ‘high start, moderate decline, high finish’ trajectory group.

Despite very similar baseline physical health scores, there was no association with any of the previously mentioned predictors and membership to the ‘high start, major decline, high finish’ trajectory group relative to the ‘high start, moderate decline, high finish’ trajectory group. In deviating to the greatest extent from baseline physical health scores, the ‘high start, major decline, high finish’ trajectory represented a subgroup of particular interest for the predictive component of this study. As baseline scores for psychosocial factors of parent pain catastrophizing, child pain catastrophizing and child trait anxiety increased, ‘high start, major decline, high finish’ trajectory membership showed a decrease in probability (Supplementary Figure 1).

#### HRQOL – psychosocial health

Membership in the ‘moderate start, high finish’ trajectory group as compared to the ‘high start, high finish’ trajectory group was associated with increased magnitude of highest curve (RRR[95% CI] = 1.06 [1.02-1.09]), higher child pain catastrophizing scores (RRR[95% CI] = 2.41 [1.52-3.83]), both child state (RRR[95% CI] = 1.97 [1.23-3.16]), and trait (RRR[95% CI] = 4.78 [2.60-8.79]) anxiety scores, as well as parent state anxiety scores (RRR[95% CI] = 1.87 [1.16-3.02]). There was no association between additional predictors with membership in the ‘moderate start, high finish’ trajectory group as compared to the ‘high start, high finish’ trajectory group.

The improvements in psychosocial health experienced by patients in the ‘moderate start, high finish’ group also constituted an area of particular interest. As the highest curve magnitude increased, the probability of belonging to the ‘moderate start, high finish’ trajectory correspondingly increased (Supplementary Figure 2). Furthermore, higher scores for child and parent pain catastrophizing, child and parent state anxiety and child trait anxiety generally indicated an increased predicted probability of ‘moderate start, high finish’ subgroup membership. The predicted probability according to parent trait anxiety scores appeared relatively unchanged.

Membership in the ‘high moderate start, high finish’ trajectory group as compared to the ‘high start, high finish’ trajectory group was associated with higher child pain catastrophizing scores (RRR[95% CI] = 2.01 [1.30-3.12]), parent pain catastrophizing scores (RRR[95% CI] = 1.58 [1.02-2.46]), both child state (RRR[95% CI] = 1.65 [1.08-2.51]), and trait (RRR[95% CI] = 5.12 [2.77-9.44]) anxiety scores, as well as parent state anxiety scores (RRR[95% CI] = 1.77 [1.16-2.71]). Remaining predictors were not associated with membership in the ‘high moderate start, high finish’ trajectory group as compared to the ‘high start, high finish’ trajectory group.

Membership in the ‘moderate start, moderate finish’ trajectory group as compared to the ‘high start, high finish’ trajectory group was associated with higher child pain catastrophizing scores (RRR[95% CI] = 2.77 [1.69-4.55]), both child state (RRR[95% CI] = 3.05 [1.76-5.28]), and trait (RRR[95% CI] = 11.19 [5.12-24.45]) anxiety scores, as well as both parent state (RRR[95% CI] = 1.68 [1.07-2.63]) and trait (RRR[95% CI] = 1.82 [1.13-2.93]) anxiety scores. There was no association between additional predictors with membership in the ‘moderate start, moderate finish’ trajectory group as compared to the ‘high start, high finish’ trajectory group.

## Discussion

This investigation explored HRQOL trajectories in patients with AIS treated surgically while examining demographic, psychosocial, and perioperative factors as possible predictors of cohort membership. The vast majority of patients in this study were within healthy baseline targets at 1-year post-surgery; however, it is of note that a considerable number of patients were within healthy baseline targets to start, suggesting minimal added health improvements owing to surgery during the first year post-operatively. This is evident in the psychosocial health trajectories, where only 1 trajectory subgroup (representing 17.5% of the study population) saw significant positive psychosocial health change postoperatively, whereas the remaining patient populations were relatively unchanged over the course of the study. The major improvement over baseline experienced by the 1 psychosocial trajectory subgroup may represent a portion of the AIS patient population who encounter increased difficulties regarding their appearance and who report greater limitations on their social interactions due to their back^28,29^. Highest curve magnitude constituted a significant predictor of membership in this particular trajectory subgroup over the ‘high start, high finish’ reference trajectory indicating unique post-operative psychosocial gains for patients undergoing surgery for more severe angles of curvature.

While all 3 physical health trajectories were within range of healthy at 1 year following surgery, this was not the case for the psychosocial health trajectories as 1 of the 4 subgroups did not attain the established healthy threshold. This suggests that fusion for AIS yields greater impact on physical health rather than psychosocial health. Despite the significant initial drops in physical HRQOL observed across trajectory groups, it is interesting to note that psychosocial HRQOL generally remained at baseline levels or higher in early recovery.

Variables relating to the patient’s prior and current medical experience such as prematurity, previous surgery, previous hospitalization and length of stay were not generally predictive of physical or psychosocial trajectory membership. One exception however was seen in the context of physical health where patients were more likely to belong to the ‘moderate start, minor decline, high finish’ trajectory group rather than the ‘high start, moderate decline, high finish’ trajectory group based on greater extent of previous hospitalizations. Given that previous hospitalizations could be attributed to a number of conditions with broad, potentially ongoing implications for physical health, association between this variable and increased likelihood of membership in the trajectory group with a lower reported baseline physical HRQOL seems reasonable.

While research assessing psychosocial predictors with respect to HRQOL is limited, the results of this study build on prior studies that identified psychosocial risk factors for postsurgical pain in children. Previous findings have shown that child^12,30-32^ and parent^15,17^ pain catastrophizing, and anxiety symptoms^13,14,16^ are associated with higher postoperative pain intensity and these factors may precipitate negative trajectory membership. Increased child pain catastrophizing in this surgical cohort could reflect actual distress in the preoperative period from pain itself or anticipated pain from upcoming operative intervention ^33^. This reality explains the association between higher child pain catastrophizing scores and membership within trajectory groups with lower baseline HRQOL in the case of both physical and psychosocial health. Given that a different PORSCHE sub-analysis has demonstrated the patient pain recovery process to span across the entire first postoperative year, it is important to consider the negative predictive implications on recovery of baseline child pain catastrophizing in the context of postsurgical pain or anticipated pain from engaging in the rehabilitation process^16,33,34^. This may be particularly relevant to the predictor relationship between higher child pain catastrophizing scores and membership in the psychosocial trajectory group that did not reach the established healthy threshold at 1 year follow-up.

The findings of this investigation have the potential to enhance the shared decision making process between patients with AIS and clinicians when deciding between surgical and nonsurgical management. This is especially true for patients with spinal curves between 40 and 50 degrees which are recognized by the Scoliosis Research Society as borderline or a “gray zone” for surgical consideration^35^. This study employed latent class growth analysis which represents a novel statistical technique for identifying HRQOL patterns of recovery. Strengths of this investigation include the use of multiple time points for data collection at 8 different care centres across Canada.

Certain limitations should be taken into account upon consideration of our findings. Particularly, this study did not account for the relationship between complication rates and HRQOL as complication related data were not included in the original methodology for the PORSCHE study. HRQOL outcomes were also only measured up to 12 months after surgery. Considering that AIS surgeon-reported rates of long-term complications lie somewhere between 5-15%^36^, the conclusions reached in this study should only be applied to HRQOL in the first postsurgical year and should not be applied to outcomes in the longer term. Interestingly, Glassman et al.’s comparison of HRQOL measures for adult patients at 1 and 2 year follow-up from surgery for lumbar degenerative scoliosis found no significant differences between no complication, minor complication and major complication groups^37^. Furthermore, although we explored psychosocial risk factors prior to surgery, resilience factors, such as effective self-coping and self-efficacy, were not assessed.

In conclusion, the majority of patients who undergo surgery for AIS achieve physical and psychosocial HRQOL scores comparable to healthy adolescents 1 year following surgery, albeit through different post-operative recovery patterns. Varying demographic, clinical and psychosocial factors were predictive of HRQOL outcomes. Importantly, these findings may assist in the management of patients living with AIS through informing surgical decision-making along with patient counselling and expectations.

## Supplemental Material

Supplemental Material - Predicting Health-Related Quality of Life Outcomes Following Major Scoliosis Surgery in Adolescents: A Latent Class Growth Analysis Supplemental Material for Predicting Health-Related Quality of Life Outcomes Following Major Scoliosis Surgery in Adolescents: A Latent Class Growth Analysis by Jack Kerr, Edward Abraham, Amanda Vandewint, Erin Bigney, Jeffrey Hebert, Eden Richardson, Dana El-Mughayyar, Jill Chorney, Ron El-Hawary, PORSCHE Study Group, Rory McPhee, and Neil Manson in Global Spine Journal
