# Supplementary material for: Predicting Health-Related Quality of Life Outcomes Following Major Scoliosis Surgery in Adolescents: A Latent Class Growth Analysis
Source: Global Spine J. 2022 Sep 20;14(3):902–13. doi: 10.1177/21925682221126451 (PMC11192129; doi:10.1177/21925682221126451)
Supplement: Supplemental Material - Predicting Health-Related Quality of Life Outcomes Following Major Scoliosis Surgery in Adolescents: A Latent Class Growth Analysis [file sj-pdf-1-gsj-10.1177_21925682221126451.pdf]

Title:(/output/Picture12.eps
Creator:(ImageMagick)
CreationDate:(2021-10-16T
CreationDate:(2021-10-16T
LanguageLevel:1

Title:(/output/Picture11.eps)
Creator:(ImageMagick)
CreationDate:(2021-10-16T1
CreationDate:(2021-10-16T1
LanguageLevel:1

Title:(/output/Picture10.eps)
Creator:(ImageMagick)
CreationDate:(2021-10-16T[illegible]
CreationDate:(2021-10-16T[illegible]
LanguageLevel:1

Title:(/output/Picture9.eps)
Creator:(ImageMagick)
CreationDate:(2021-10-16T[illegible]
CreationDate:(2021-10-16T[illegible]
LanguageLevel:1

Title:(/output/Picture8.eps)
Creator:(ImageMagick)
CreationDate:(2021-10-16T1
CreationDate:(2021-10-16T1
LanguageLevel:1

Title:(/output/Picture6.eps)
Creator:(ImageMagick)
CreationDate:(2021-10-16T1
CreationDate:(2021-10-16T1
LanguageLevel:1

Title:(/output/Picture5.eps)
Creator:(ImageMagick)
CreationDate:(2021-10-16T[illegible]
CreationDate:(2021-10-16T[illegible]
LanguageLevel:1

Title:(/output/Picture4.eps)
Creator:(ImageMagick)
CreationDate:(2021-10-16T1[illegible]
CreationDate:(2021-10-16T1[illegible]
LanguageLevel:1

Title:(/output/Picture3.eps)
Creator:(ImageMagick)
CreationDate:(2021-10-16T
CreationDate:(2021-10-16T
LanguageLevel:1
